# Clinical Significance and Revisiting the Meaning of CA 19-9 Blood Level Before and After the Treatment of Pancreatic Ductal Adenocarcinoma: Analysis of 1,446 Patients from the Pancreatic Cancer Cohort in a Single Institution

**DOI:** 10.1371/journal.pone.0078977

**Published:** 2013-11-08

**Authors:** Joo Kyung Park, Woo Hyun Paik, Ji Kon Ryu, Yong-Tae Kim, Youn Joo Kim, Jaihwan Kim, Byeong Jun Song, Jin Myung Park, Yong Bum Yoon

**Affiliations:** Departments of Internal Medicine and Liver Research Institute, Seoul National University College of Medicine, Seoul, Korea; Technische Universität München, Germany

## Abstract

**Background:**

Life expectancy of pancreatic ductal adenocarcinoma (PDAC) patients is usually short and selection of the most appropriate treatment is crucial. The aim of this study was to investigate the usefulness of serum CA 19-9 as a surrogate marker under no impress excluding other factors affecting CA 19-9 level other than tumor itself.

**Methods:**

We recruited 1,446 patients with PDACs and patients with Lewis antigen both negative or obstructive jaundice were excluded to eliminate the false effects on CA 19-9 level. The clinicopathologic factors were reviewed including initial and post-treatment CA 19-9, and statistical analysis was done to evaluate the association of clinicopathologic factors with overall survival (OS).

**Results:**

The total of 944 patients was enrolled, and205 patients (22%) underwent operation with curative intention and 541 patients (57%) received chemotherapy and/or radiotherapy. The median CA 19-9 levels of initial and post-treatment were 670 IU/ml and 147 IU/ml respectively. The prognostic factors affecting OS were performance status, AJCC stage and post-treatment CA 19-9 level in multivariate analysis. Subgroup analysis was done for the patients who underwent R0 and R1 resection, and patients with normalized post-operative CA 19-9 (≤37 IU/mL) had significantly longer OS and DFS regardless of initial CA 19-9 level; 32 vs. 18 months, *P*<0.001, 16 vs. 9 months, *P = *0.004 respectively.

**Conclusions:**

Post-treatment CA 19-9 and normalized post-operative CA 19-9 (R0 and R1 resected tumors) were independent factors associated with OS and DFS, however, initial CA 19-9 level was not statistically significant in multivariate analysis.

## Introduction

Pancreatic ductal adenocarcinoma (PDAC) has poor prognosis with only 6% 5-year survival rate after its diagnosis [1]. Surgical resection is the only chance of potentially curative treatment in PDAC, however only 15% of patients could be candidate for resection [2], [3]. Even after complete resection, prognosis remains poor in spite of recent improvements of surgical management and adjuvant therapy [4]. It would be explained by late stage presentation, lack of effective treatments, early recurrence and absence of clinically useful biomarker which can detect PDAC in its precursor form or earliest stages [5]. There have been many attempts to overcome such a tragic disease in many different approaches such as prevention, early detection and finding effective treatment. Despite of advances and efforts to above all, PDAC remains still an incurable disease for most patients [6]. Therefore, selection of patients with excellent response to treatment and making an accurate prediction of treatment response could be essential in management of patients with PDACs. Recently vast number of biomarkers in PDACs has been proposed, however, none of those biomarkers reported to be as useful as CA 19-9 so far [7]–[11]. Serum CA 19-9 is a mostly widely used tumor-associated antigen and it can provide useful diagnostic information in patients with PDACs. Its sensitivity and specificity are approximately 70% and 80%, respectively [12]. There are also additional roles of CA 19-9 in management of PDACs and the following roles have been discussed in many studies; pre- and post- operative level in resectable PDACs, base line level in advanced PDACs and post-treatment level as a potential marker for evaluation of treatment response [13]. Although, CA 19-9 is one of the most well known and the oldest biomarker in PDACs, its efficacy for predicting prognosis and monitoring patients remains controversial [14]–[16]. CA 19-9 level can be affected in patients with obstructive jaundice and also there is 7% to 10% of the population with the Lewis phenotype Le (a - b -) meaning CA 19-9 non-expressers [17]. Furthermore, many of the previous studies enrolled the patients with jaundice or did not exclude the patients with negative for both Lewis A and B antigens [18]–[24]. Therefore, the aim of this study was to investigate the true meaning and the usefulness of CA 19-9 as a prognostic marker in treatment of PDACs excluding other factors affecting the level of CA 19-9 other than tumor itself.

## Materials and Methods

### Ethics Statement

Based on the Declaration of Helsinki, Institutional Review Board of Seoul National University Hospital approved the retrospective use of all the corresponding data for the present study (IRB Approval Number: H-1110-099-382). Data were obtained retrospectively from electronic medical records, and all of them were documented according to coded number for anonymity.

### Patients

Newly diagnosed PDAC patients were enrolled from the pancreatic cancer cohort registry in Seoul National University Hospital, a tertiary teaching hospital from January 1996 to July 2011. The total of 1,572 patients was initially enrolled from the pancreatic cancer cohort registry, and patients with lost follow-ups and insufficient data were excluded from the study (Figure S1 in File S1). The following exclusion criteria was applied to the 1,446 patients with PDACs: 1) patients with CA 19-9 level less than 5 U/mL at the time of diagnosis as they were considered to be CA 19-9 nonsecretors’ group (negative for both Lewis A and B antigens) [4], 2) patients with total bilirubin level more than 2 mg/dL at the time of diagnosis as obstructive jaundice would affect the serum level of CA 19-9. The last 944 patients were enrolled to the final entry of this study.

### Acquisition of Clinical Data and Measurement of Serum CA 19-9

Clinical data was collected based on electronic medical records, case report form of cohort registry and follow-up phone calls: age, gender, performance status, time of diagnosis, AJCC TNM cancer stage (American Joint Committee on Cancer (AJCC) 7^th^ edition), laboratory findings, type of treatment, pathologic reports, overall survival (OS) and disease-free survival (DFS). Serum CA 19-9 was measured by radioimmunoassay and upper limit of normal was 37 U/mL. Initial CA 19-9 level was measured at the time of diagnosis and post-treatment CA 19-9 level was defined as the measurement of CA 19-9 level after any kinds of treatment such as resection with curative intention, chemotherapy, concurrent chemoradiation therapy or radiotherapy. Also, CA 19-9 was measured regularly during the clinical course of treatment with 3 to 6 months of interval afterwards.

### Standard Treatment and Follow-Up of Patients with PDACs

The study patients were treated according to standard treatment protocol in our institution and it was described in Figure S2 in File S1. Operable PDACs underwent resection with curative intention and adjuvant therapy was done unless patients were in poor performance status or didn’t want any further treatment. Unresectable PDACs were treated with chemotherapy and/or radiation therapy or best supportive care only. Therefore, initial treatment was defined as the following three different groups and they are the operation group (resection with curative intention only), chemotherapy and/or radiotherapy group and the best supportive care group. In addition, any kinds of palliative operation and radiation therapy for distant metastasis such as bone metastasis rather than a local control of primary PDACs were considered as a part of best supportive care and it was not counted as an initial treatment. If obstructive jaundice developed during the course of treatment and follow-up period, chemotherapy and radiotherapy were discontinued and immediate palliation of obstructive jaundice was done because obstructive jaundice and combined acute cholangitis could be a life-threatening condition.

### Treatment Response and Survival Data

Tumor response after chemotherapy and/or radiation therapy was assessed by RECIST criteria [25]. Disease control rate was defined as the total rates of complete response, partial response and stable disease after treatment. OS was defined as the length of time after the initial diagnosis of PDAC to time of death as a result of any cause. DFS was applied only to the PDACs with curative resection and defined as the length of time after radical resection until the first progression or death as a result of any cause, if disease progression did not occur based on simple and standardized radiographic imaging studies.

### Statistical Analysis

We dichotomized the patients according the CA 19-9 level, and compared the clinical factors between the 2 groups with χ^2^ test and Student *t* test. OS and DFS with associated variables were calculated by the Kaplan-Meier method from log-rank test. Multivariate analysis was performed on factors with associated *P* values of <0.2 by univariate analysis and Cox proportional hazard model was used in multivariate analysis to identify independent factors associated with OS and DFS. A *P* value of <0.05 was considered to be statistically significant. All statistical analysis was performed using SPSS v.18.0 (IBM Corp., Armonk, NY, USA).

## Results

### Baseline Demographics and Clinical Characteristics

The median age of study patients was 63 (range 29 to 96) years old and there were 587 males (62%) and 357 females (38%) and male to female ratio was 1.6. Most of the patients (841 patients, 86%) were in ECOG performance status 0 or 1 and distribution of AJCC clinical staging was as follows: 8 patients (IA, IB), 196 patients (IIA, IIB), 252 patients (III) and 488 patients (IV). The number of patients who underwent operation with curative intent was approximately 205 (22%) and patients with chemotherapy and/or radiotherapy were 541 (57%). The median serum CA 19-9 level at the time of diagnosis was 670 (range 5–1,119,000) U/mL and the following clinical parameters were compared between the two groups: age, sex, AJCC stage, performance status and type of initial treatment (Table 1). The patients with better performance status, lower AJCC stage and operation group were significantly associated with lower serum CA 19-9 level (<670 U/mL, median). Therefore, initial CA 19-9 level at the time of diagnosis significantly associated with clinical stage and type of initial treatment, both of which reflect the operability of PDACs (Table 1).

**Table 1:** Baseline demographics and clinical characteristics.

Variable	CA 19-9<670 (U/mL) (n = 472)	CA 19-9≥670 (U/mL) (n = 472)	*P*
Gender			.46
Male	299	184	
Female	173	884	
Age	63.3	62.8	.50
Performance status (ECOG)			.009
0, 1	420	392	
2 to 4	52	80	
**AJCC stage**			**<.001**
IA	6	0	
IB	2	0	
IIA	72	26	
IIB	75	23	
III	131	121	
IV	186	302	
**Initial treatment**			**<.001**
Operation	153	52	
Chemo- and/or radiotherapy	230	311	
Best supportive care	89	109	

### Initial Serum CA 19-9 Level and its Significance

**Figure 1:**
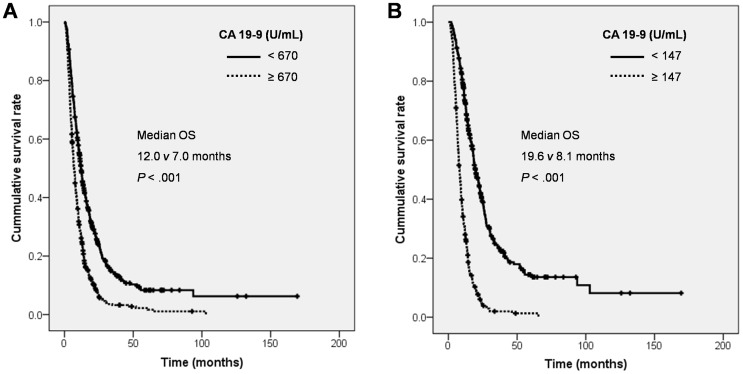
Kaplan-Meier curve of OS according to CA 19-9 level in various clinical courses. (A) Initial CA 19-9 with median cut off value and (B) post-treatment CA 19-9 with median cut off value. Abbreviations: OS, overall survival.

Initial CA 19-9 level was measured in the total of 944 patients who were enrolled in this study at the time of diagnosis, and the median level was 670 U/mL. The mean CA 19-9 level with standard deviation was 14511±75120 U/mL. The mean adjusted CA 19-9 level (CA 19-9 divided by total bilirubin level) with standard deviation was 18190±79919. The patients whose initial CA 19-9 level was below 670 U/mL had 12.0 months of median OS while patients with CA 19-9 level more than 670 U/mL had 7.0 months of median OS and it was statistically significant (*P*<0.001, Figure 1A). Again, we mentioned above in method section that the patients without jaundice (≤2 mg/dL) were only included in this study to exclude one of the most strong factor affecting the level of CA 19-9 such as obstructive jaundice. Therefore, we could observe initial CA 19-9 level was significantly associated with OS in PDAC patients.

### Post-Treatment Serum CA 19-9 Level and its Significance

Post-treatment serum CA 19-9 level in 746 patients who underwent initial treatment such as operation or chemo-and/or radiotherapy (except for best supportive care group) was analyzed. CA 19-9 level decreased more than half in 395 patients (53%) after treatment: 152 patients (74%) after operation and 233 patients (43%) after chemotherapy and/or radiotherapy. The post-treatment serum CA 19-9 level was ranged from 0 to 947,000 (median 147) U/mL and OS was significantly different according to post-treatment CA 19-9 level; median OS: 19.6 vs. 8.1 months, *P*<0.001 (Figure 1B). In other words, the patients whose post-treatment CA 19-9 level less than its median value had 19.6 months of median OS and others had 8.1 months of median OS (*P*<0.001).

### Subgroup Analyses: Treatment Response in Unresectable PDACs

The total of 739 patients who were unable to go through curative resection was further analyzed to find out the post-treatment serum CA 19-9. The decrement of post-treatment serum CA 19-9 level was significantly correlated with disease control rate among the patients who received chemotherapy and/or radiation therapy (87% vs. 37%, *P*<0.001). Treatment response was evaluated in chemotherapy and/or radiation therapy groups and there were 2 patients with complete remission (CR) after palliative chemotherapy. One patient with liver metastasis had CR after 9 cycles of gemcitabine and cisplatin combination therapy and it lasted 7.0 months after CR. Initial and post-treatment serum CA 19-9 level of the patient was 10.0 and 5.0 U/mL, respectively. The other patient also had liver metastasis at the time of diagnosis and received 6 cycles of gemcitabine and capecitabine combination therapy and it lasted 5.7 months after CR. Initial and post-treatment serum CA 19-9 level of the patient was 17.4 and 1.1 U/mL, respectively. Partial response and stable disease response appeared in 81 (17%) and 167 (36%) patients respectively.

### Subgroup Analyses: Treatment Response in R0 and R1 Resected PDACs

**Figure 2:**
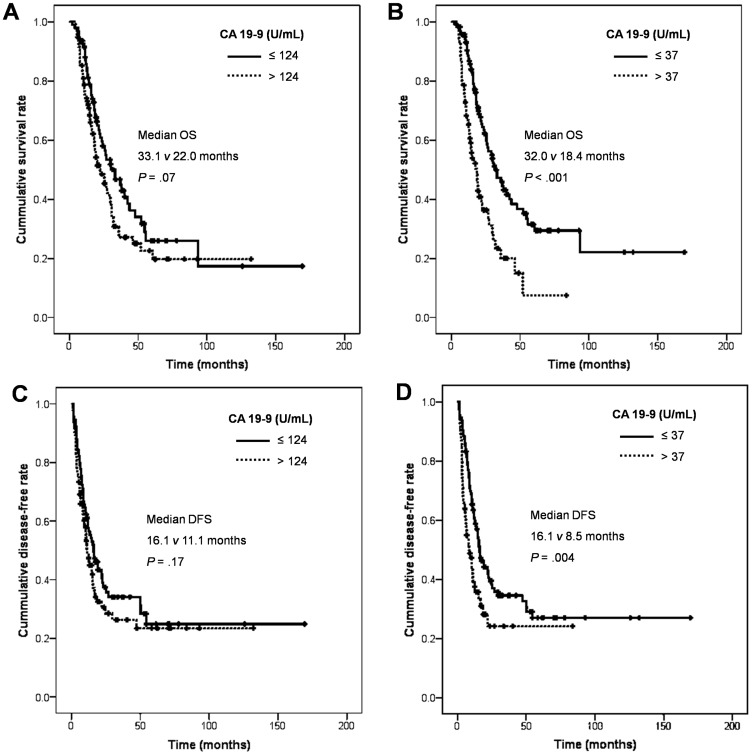
Kaplan-Meier curve of OS and DFS according to CA 19-9 level in R0 and R1 resection. (A, C) Initial CA 19-9 with median cut off value and (B, D) post-operative CA 19-9 with normal cut off value. Abbreviations: OS, overall survival; DFS, disease-free survival.

The total of 189 patients who underwent operation with curative intention (R0 and R1 resection only) was further analyzed to find out significance of serum CA 19-9. There were 156 patients with R0 resection and 33 patients with R1 resection. The median OS was 26.8 (95% CI, 21.2 to 32.4) months and the total of 112 patients (59%) had died of any cause (Figure S3A in File S1). The significant clinicopathologic factors associated with OS were also analyzed and they are the followings; N stage, resection margin of surgical specimen, angiolymphatic and venous invasion from the pathologic evaluation of surgical specimens, adjuvant treatment and post-operative normalized serum CA 19-9 (Table S1 in File S1). Interestingly, initial CA 19-9 level did not affect OS while patients with normalized CA 19-9 level after the resection had significantly longer OS no matter what their initial CA 19-9 level was: OS 32.0 vs. 18.4 months, *P*<0.001 (Figure 2A and B). In multivariate analysis, post-operative serum CA 19-9 level, resection margin, angiolymphatic invasion, venous invasion and adjuvant treatment were independent factors affecting OS of curatively resected PDACs (R0 and R1 resection only, Table 2).

**Table 2:** Prognostic factors affecting overall survival in R0 and R1 resection by multivariate analysis.

Variable	HR (95% CI)	*P*
T stage	1.73 (0.74 to 4.02)	.20
N stage	1.04 (0.67 to 1.62)	.88
Initial CA 19-9>124 U/mL	0.90 (0.55 to 1.47)	.66
**Post-operative CA 19-9>37 U/mL**	**1.96 (1.16 to 3.31)**	**.01**
**R1 resection**	**1.84 (1.14 to 2.95)**	**.01**
**Angiolymphatic invasion**	**1.70 (1.10 to 2.61)**	**.02**
**Venous invasion**	**2.16 (1.33 to 3.51)**	**.002**
Perineural invasion	1.07 (0.61 to 1.88)	.81
**Adjuvant treatment**	**0.52 (0.33 to 0.83)**	**.006**

The median DFS was 14.0 (95% CI, 10.8 to 17.2) months and the total of 121 patients (66%) had recurrence of disease after the radical resection (Figure S3B in File S1). The significant clinicopathologic factors associated with recurrence were the followings; T, N stage, resection margin of surgical specimen, angiolymphatic, perineural and venous invasion from the pathologic evaluation of surgical specimens and post-operative normalized serum CA 19-9 (Table S2 in File S1). Also, initial CA 19-9 level did not affect DFS whereas patients with normalized CA 19-9 level after the resection had significantly longer DFS regardless of their initial CA 19-9 level: DFS 16.1 vs. 8.5 months, *P* = 0.004 (Figure 2C and D). In multivariate analysis, post-operative serum CA 19-9 level and venous invasion were independent factors affecting DFS of curatively resected PDACs (R0 and R1 resection only, Table 3).

**Table 3:** Prognostic factors affecting disease-free survival in R0 and R1 resection by multivariate analysis.

Variable	HR (95% CI)	*P*
T stage	1.77 (0.75 to 4.20)	.19
N stage	1.14 (0.74 to 1.76)	.57
Initial CA 19-9>124 U/mL	0.96 (0.61 to 1.51)	.86
**Post-operative CA 19-9>37 U/mL**	**1.93 (1.20 to 3.08)**	**.006**
R1 resection	1.53 (0.96 to 2.46)	.08
Angiolymphatic invasion	1.46 (0.95 to 2.24)	.09
**Venous invasion**	**2.20 (1.40 to 3.46)**	**.001**
Perineural invasion	0.95 (0.55 to 1.62)	.84

### Prognostic Factors Affecting Overall Survival Rate

The median OS of the study patients was 9.1 (95% CI, 8.4 to 9.8) months and 822 out of 944 study patients (87%) died during the follow-up. The clinicopathologic parameters were analyzed and the following factors were significantly associated with OS in univariate analysis: age, gender, performance status, AJCC stage, initial serum CA 19-9 level and post-treatment serum CA 19-9 level (Table S3 in File S1). We also analyzed above factors in multivariate analysis and patients with better performance status, lower clinical stage and lower post-treatment CA 19-9 were the independent prognostic factors associated with better OS (Table 4). However, initial CA 19-9 level was not statistically significant factor in predicting OS from multivariate analysis.

**Table 4:** Prognostic factors affecting overall survival by multivariate analysis.

Variable	HR (95% CI)	*P*
Age >65 years	1.14 (0.96 to 1.36)	.14
Male	1.18 (0.98 to 1.41)	.08
**Performance status** **(ECOG)**	**1.42 (1.14 to 1.78)**	**.002**
**AJCC stage**	**1.81 (1.60 to 2.05)**	**<.001**
Initial CA 19-9≥670 U/mL	0.95 (0.76 to 1.18)	.65
**Post-treatment CA 19-9**≥**147 U/mL**	**2.50 (1.98 to 3.17)**	**<.001**

## Discussion

CA 19-9 is one of the oldest biomarker used in patients with PDACs and its usefulness and clinical significance have been reported in many studies. Unfortunately, there are still other factors affecting the serum CA 19-9 level other than PDAC itself and it always makes it unclear and brings limitations in interpretation of the clinical course [15], [26]–[29]. There have been many investigations to develop a surrogate biomarker capable of detecting, monitoring and predicting the prognosis of PDACs [7], [11], [30]. There is no other excellent marker than CA 19-9 in terms of sensitivity and specificity even though it still has many flaws [15], [31]–[34]. Therefore, it is very important to re-evaluate the clinical usefulness of CA 19-9 as a surrogate biomarker in patients with PDACs. This study was designed for the first time to our knowledge that other factors responsible for affecting serum CA 19-9 level (obstructive jaundice and CA 19-9 nonsecretors) were excluded, and recruited one of the largest number of PDAC cohort from the single tertiary referral hospital. As a result, there were 944 patients eligible to this study and their clinicopathologic factors including CA 19-9 level was evaluated whether they have statistically significant association with overall survival of the patients. At first, patients with below the median level of initial CA 19-9 and post-treatment CA 19-9 were significantly associated with longer OS in univariate analyses. However, post-treatment CA 19-9 and some obvious clinical factors such as AJCC staging and performance status were remained as independent prognostic factors associated with OS in multivariate analyses. It was interesting to look into the fact that we applied the exclusion criteria to the 1,446 PDACs if they were CA 19-9 nonsecretors with negative for both Lewis A and B antigens and had obstructive jaundice, but still initial CA 19-9 level did not have significant effect over survival of the patients with PDACs. In addition, subgroup analyses was done among the patients with curative resection (R0 and R1 only) and patients with normalized post-operative CA 19-9 (≤37 IU/mL) had significantly longer DFS and OS regardless of initial CA 19-9 level; 16 vs. 9 months, *P = *0.004, 32 vs. 18 months, *P*<0.001 respectively. In addition, other clinicopathologic factors such as resection margin status (R0 vs. R1), angiolymphatic invasion, venous invasion and adjuvant treatment were independent factors associated with OS in multivariate analyses. Glen and his colleagues also have showed post-operative CA 19-9 as well as post-treatment CA 19-9 level has a great deal of utilities in evaluation of clinical courses of PDACs [16]. Also, there have been several studies supporting our results that the certain amount of decrease in CA 19-9 (>15% to 30%) during chemotherapy was the only independent predictor of survival in locally advanced and metastatic PDACs [21], [22], [35]. On the contrary to our data, Hess et al. reported that pretreatment serum CA 19-9 concentration was an independent prognostic factor for survival, but a decrease in concentration during chemotherapy was not significantly associated with lengthened survival compared with those who did not have a corresponding decrease [13]. Moreover, some other studies also have reported that initial CA 19-9 level or preoperative CA 19-9 was significant predictive biomarker along with or without post-treatment CA 19-9 level during the treatments of PDACs [4], [31], [36]–[38]. However, none of above studies have stratified other factors might be partially responsible for altering CA 19-9 blood level rather than PDAC itself.

To investigate and find out true meaning of CA 19-9 as a biomarker in patients with PDACs is based on the hypothesis that serum CA 19-9 reflects the potential tumor burden of the patients beyond the evidence from imaging studies or microscopic examination. As a result, we showed the post-treatment CA 19-9 level (< median) or normalized post-operative CA 19-9 level were independently associated with longer survival of the patients. We would like to address a few things regarding clinical significance of CA 19-9 from our study that CA 19-9 is one of noninvasive blood biomarkers and may be ideal for prediction of prognosis and evaluating treatment response in patients with PDACs. Post-treatment CA 19-9 (including operation) level was significantly associated with DFS and OS while initial CA 19-9 level or preoperative CA 19-9 level did not have clinical significance. However, we still could not find out reasonable explanation why initial CA 19-9 blood level did not meet the clinical significance even though we excluded the patients with false increase or decrease of CA 19-9 level. But so far, other candidate biomarkers including CA 19-9 to accurately detect tumors or predict prognosis have not emerged yet. In other words, it is essential for PDAC biomarker to play a role in selection of patients to treat them until they have an optimal response associated with longer survival.

## Conclusions

We studied clinical significances of serum CA 19-9 in patients with PDACs that post-treatment CA 19-9 and normalized post-operative CA 19-9 were the independent prognostic markers for DFS and OS, and ultimately we might want to say it is a surrogate biomarker in evaluating treatment response of PDACs. Therefore, further investigations of CA 19-9 concentration would make us possible to understand its prognostic role in PDAC progression and to use it as a noninvasive biomarker in accurate selection of PDACs for further treatment.

## Supporting Information

File S1 Supporting information.
(DOCX)
