# Supplementary material for: Clinical Significance and Revisiting the Meaning of CA 19-9 Blood Level Before and After the Treatment of Pancreatic Ductal Adenocarcinoma: Analysis of 1,446 Patients from the Pancreatic Cancer Cohort in a Single Institution
Source: PLoS One. 2013 Nov 8;8(11):e78977. doi: 10.1371/journal.pone.0078977 (PMC3826753; doi:10.1371/journal.pone.0078977)
Supplement: File S1 — Supporting information. (DOCX) [file pone.0078977.s001.docx]

**Combined Supporting Information Files**

**
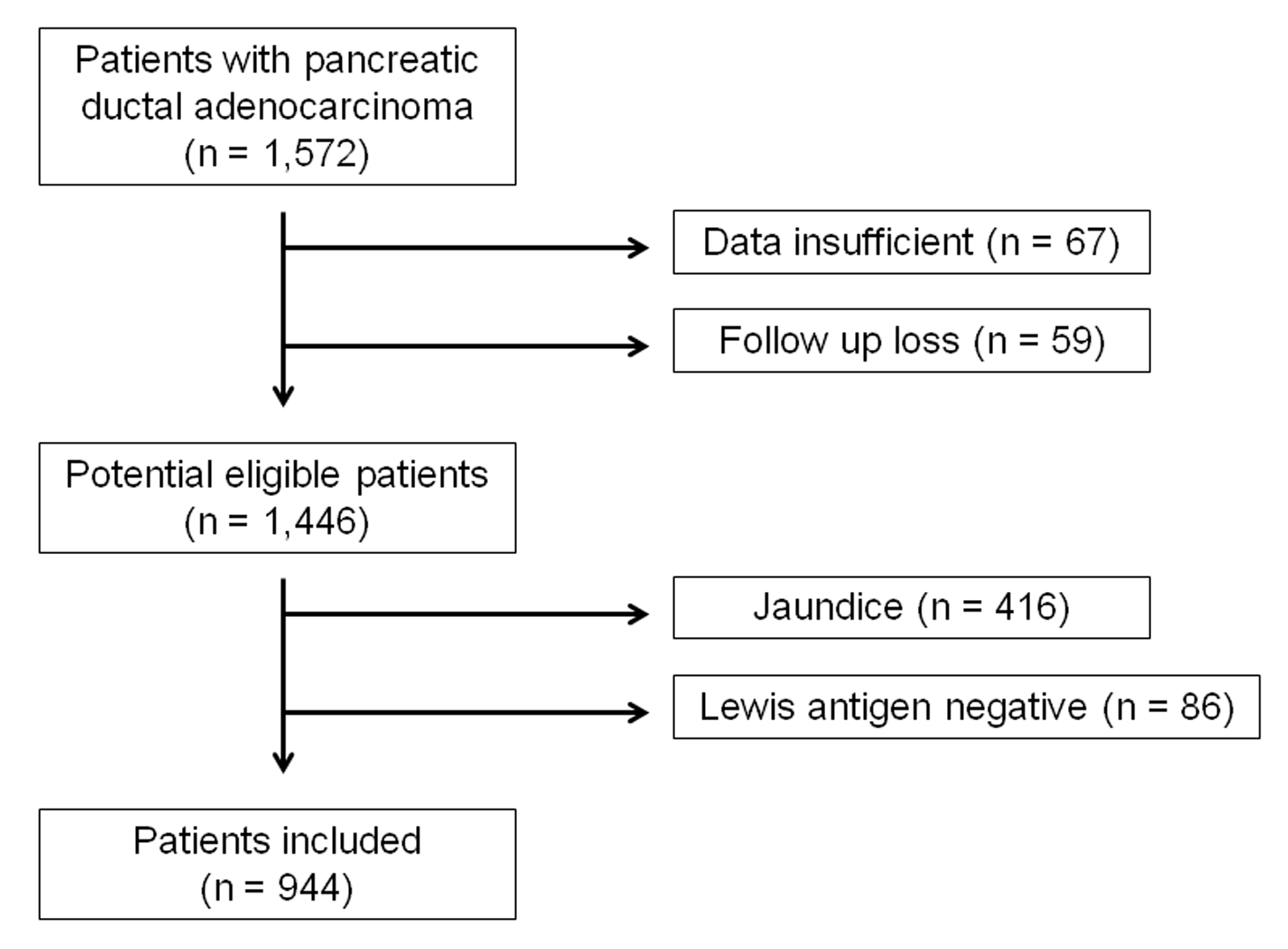
**

**Figure S1.** Cohort of study patients.

**
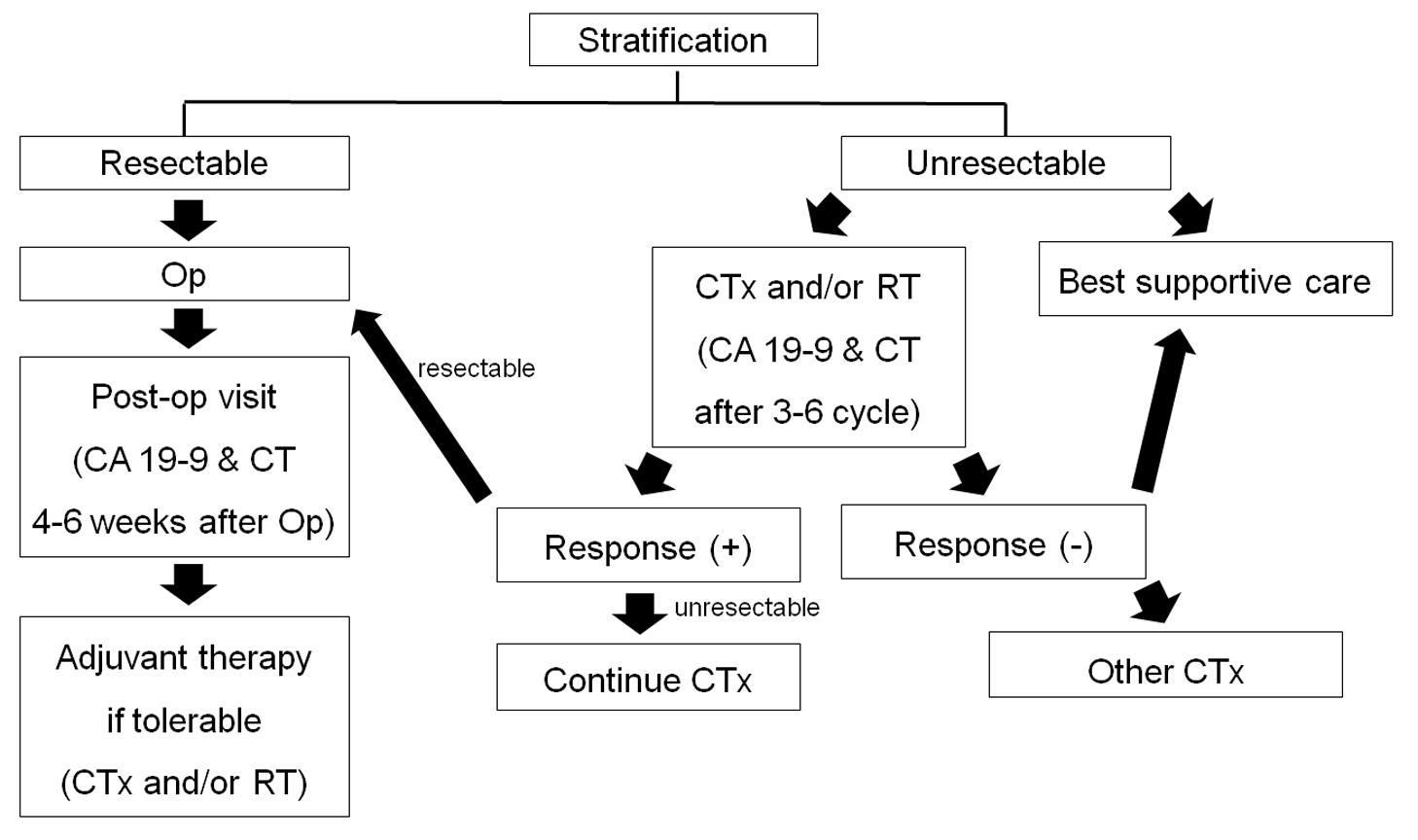
**

**Figure S2.** Standard treatment and follow-up schedule for patients with pancreatic ductal adenocarcinoma.

Abbreviations: Op, operation; CTx, chemotherapy; RT, radiation therapy.

**
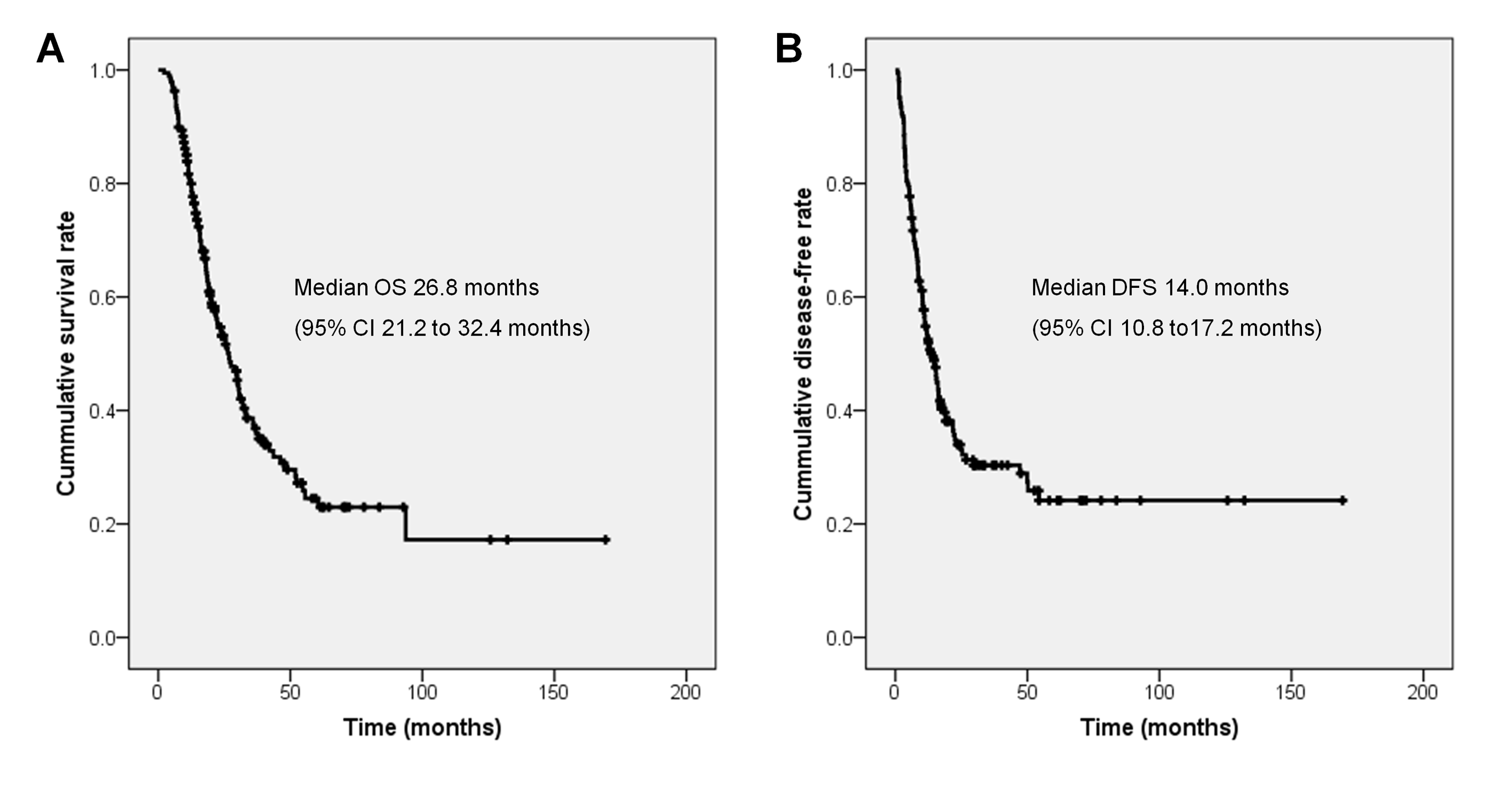
**

**Figure S3.** Kaplan-Meier curve of (A) OS and (B) DFS in R0 and R1 resected pancreatic ductal adenocarcinoma.

Abbreviations: OS, overall survival; DFS, disease-free survival.

**Table S1.Prognostic factors affecting overall survival in R0 and R1 resection by univariate analysis.**

| **Variable** | **Median overall survival**  **(95% CI, months)** | ***P*** |
| --- | --- | --- |
| Age (years) |  | .38 |
| ≤ 65 (n = 95) | 27.5 (17.7 to 37.3) |  |
| > 65 (n = 94) | 26.8 (20.2 to 33.4) |  |
| Gender |  | .65 |
| Male (n = 99) | 29.0 (18.4 to 39.6) |  |
| Female (n = 90) | 25.9 (20.9 to 30.9) |  |
| T stage |  | .09 |
| 1, 2 (n = 8) | - |  |
| 3, 4 (n = 181) | 25.9 (20.1 to 31.7) |  |
| **N stage** |  | **.02** |
| 0 (n = 95) | 30.6 (22.2 to 39.0) |  |
| 1 (n = 94) | 18.8 (13.7 to 23.9) |  |
| Initial CA 19-9 (median, U/mL) |  | .07 |
| ≤ 124 (n = 94) | 33.1 (22.2 to 44.0) |  |
| > 124 (n = 95) | 22.0 (14.9 to 29.1) |  |
| **Post-operative CA 19-9** (U/mL) |  | **< .001** |
| ≤ 37 (n = 117) | 32.0 (23.3 to 40.7) |  |
| > 37 (n = 70) | 18.4 (13.3 to 23.5) |  |
| **Resection margin** |  | **.001** |
| R0 (n = 156) | 29.0 (23.6 to 34.4) |  |
| R1 (n = 33) | 16.2 (10.3 to 22.1) |  |
| Tumor differentiation |  | .07 |
| Well (n = 10) | 93.7 (-) |  |
| Moderate (n = 147) | 22.6 (16.9 to 28.3) |  |
| Poor (n = 8) | 26.8 (0 to 53.9) |  |
| **Angiolymphatic invasion** |  | **.001** |
| Y (n = 69) | 17.9 (13.8 to 22.0) |  |
| N (n = 118) | 30.6 (25.4 to 35.8) |  |
| **Venous invasion** |  | **< .001** |
| Y (n = 40) | 14.4 (11.1 to 17.7) |  |
| N (n = 147) | 30.7 (23.7 to 37.7) |  |
| Perineural invasion |  | .08 |
| Y (n = 145) | 22.6 (16.3 to 28.9) |  |
| N (n = 41) | 35.9 (22.7 to 49.1) |  |
| **Adjuvant treatment** |  | **.03** |
| Y (n = 139) | 30.6 (22.0 to 39.2) |  |
| N (n = 44) | 21.4 (16.6 to 26.2) |  |

**Table S2. Prognostic factors affecting disease-free survival in R0 and R1 resection by univariate analysis.**

| **Variable** | **Disease-free survival (95% CI, months)** | ***P*** |
| --- | --- | --- |
| Age (years) |  | .06 |
| ≤ 65 (n = 93) | 10.3 (6.1 to 14.5) |  |
| > 65 (n = 91) | 16.3 (11.3 to 21.3) |  |
| Gender |  | .41 |
| Male (n = 96) | 14.7 (9.8 to 19.6) |  |
| Female (n = 88) | 13.3 (9.9 to 16.7) |  |
| **T stage** |  | **.047** |
| 1, 2 (n = 7) | - |  |
| 3, 4 (n = 177) | 12.6 (9.5 to 15.7) |  |
| **N stage** |  | **.004** |
| 0 (n = 94) | 18.5 (13.0 to 24.0) |  |
| 1 (n = 90) | 10.2 (8.1 to 12.3) |  |
| Initial CA 19-9 (median, U/mL) |  | .17 |
| ≤ 124 (n = 90) | 16.1 (11.2 to 21.0) |  |
| > 124 (n = 94) | 11.1 (8.6 to 13.6) |  |
| **Post-operative CA 19-9** (U/mL) |  | **.004** |
| ≤ 37 (n = 113) | 16.1 (12.6 to 19.6) |  |
| > 37 (n = 69) | 8.5 (5.0 to 12.0) |  |
| **Resection margin** |  | **< .001** |
| R0 (n = 154) | 15.6 (12.2 to 19.0) |  |
| R1 (n = 30) | 6.2 (1.1 to 11.3) |  |
| Tumor differentiation |  | .20 |
| Well (n = 10) | 16.7 (0 to 60.5) |  |
| Moderate (n = 144) | 12.4 (9.3 to 15.5) |  |
| Poor (n = 7) | 5.8 (0.7 to 10.9) |  |
| **Angiolymphatic invasion** |  | **< .001** |
| Y (n = 67) | 8.7 (7.3 to 10.1) |  |
| N (n = 115) | 18.5 (11.8 to 25.2) |  |
| **Venous invasion** |  | **< .001** |
| Y (n = 39) | 6.2 (4.6 to 7.8) |  |
| N (n = 143) | 16.7 (11.5 to 21.9) |  |
| **Perineural invasion** |  | **.008** |
| Y (n = 142) | 12.4 (9.3 to 15.5) |  |
| N (n = 39) | - |  |
| Adjuvant treatment |  | .19 |
| Y (n = 137) | 14.7 (11.5 to 17.9) |  |
| N (n = 44) | 10.5 (4.7 to 16.3) |  |

**Table S3. Prognostic factors affecting overall survival by univariateanalysis.**

| **Variable** | **Median overall survival (95% CI, months)** | ***P*** |
| --- | --- | --- |
| **Age** (median, years) |  | **.04** |
| ≤ 65 (n = 492) | 10.1 (9.3 to11.0) |  |
| > 65 (n = 452) | 8.1 (7.2 to 9.0) |  |
| **Gender** |  | **.04** |
| Male (n = 587) | 8.9 (8.0 to 9.8) |  |
| Female (n = 357) | 9.9 (8.7 to 11.3) |  |
| **Performance status** (ECOG) |  | **< .001** |
| 0, 1 (n = 812) | 10.1 (9.3 to 10.9) |  |
| 2 to 4 (n = 132) | 3.9 (3.0 to 4.8) |  |
| **AJCC stage** |  | **< .001** |
| I (n = 8) | - |  |
| II (n = 196) | 22.3 (17.6 to 27.0) |  |
| III (n = 252) | 10.7 (9.4 to 12.0) |  |
| IV (n = 488) | 5.8 (5.3 to 6.3) |  |
| **Initial CA 19-9** (median, U/mL) |  | **< .001** |
| < 670 (n = 472) | 12.0 (10.8 to 13-2) |  |
| ≥ 670 (n = 472) | 7.0 (6.4 to 7.6) |  |
| **Post-treatment CA 19-9** (median, U/mL) |  | **< .001** |
| < 147 (n = 319) | 19.6 (17.2 to 22.0) |  |
| ≥ 147 (n = 320) | 8.1(7.3 to 8.9) |  |
